# From early detection to rehabilitation in the community: reading beyond the blog testimonies of survivors’ quality of life and prostate cancer representation

**DOI:** 10.1186/s12955-016-0568-6

**Published:** 2016-12-16

**Authors:** Margareth Santos Zanchetta, Marguerite Cognet, Mary Rachel Lam-Kin-Teng, Marie Elisabeth Dumitriu, Lise Renaud, Jacques Rhéaume

**Affiliations:** 1 Ryerson University- Faculty of Community Services, Daphne Cockwell School of Nursing, 350 Victoria St. office POD 470C, Toronto, ON M5B 2K3 Canada; 2 Université Denis Diderot- UFR Sciences sociales, Unité de recherche Migrations et Sociétés, Paris, France; 3 Université du Québec à Montréal, CSSS de la Montagne, Ministère de la santé et des services sociaux du Québec, Montréal, Québec Canada; 4 CSSS de la Montagne, Ministère de la santé et des services sociaux du Québec, Montréal, Québec Canada

## Abstract

**Background:**

Survivors’ testimonies can reveal much about men’s experiences of prostate cancer and impacts on their quality of life (QOL) during the clinical trajectory of the disease. These survivors’ shared thoughts and views were hypothesized to reflect salient features of their lived social representation of prostate cancer.

**Context:**

We explored the content of testimonies posted by men to a public blog hosted by a French national prostate cancer patients’ association. The study question, “What do French bloggers’ testimonies reveal about their lived experiences with prostate cancer, especially regarding their quality of life in community settings, that underpin their social representation of prostate cancer?” guided the exploration and analysis of the textual data. The aims were to better understand men’s experiences and predominant thoughts and views, to elucidate patients’ behaviours, and to enlighten medical policy and practice.

**Purpose:**

Explore issues of QOL as reported by French prostate cancer survivors in a public blog by: (a) identifying the salient aspects and issues of the experience of living with prostate cancer from the perspective of survivors; and (b) analyzing the content in the posted testimonies regarding perceived and lived impacts of prostate cancer on QOL.

**Methods:**

A critical ethnographic study guided the selection of textual data from 196 male bloggers’ testimonies about prostate cancer posted in the period from 2008 to 2013. Media content analysis method was undertaken on blog testimonies, framed by a multidimensional conceptual framework of QOL.

**Results:**

Testimonies focused mainly on medical care and rehabilitation, recovery, health education and self-care, as well as on a global vision of prostate cancer and its impacts on personal views of manhood and masculinity. The language used indicated that political, educative and compassionate discourses were intertwined to create a complex representation of the experience and effects of prostate cancer; this multi-faceted representation can inform the public and professional debate about men’s capacity to provide emotional support and problem-solve within a community of interest.

**Conclusion:**

Findings, while based on data limited to mostly one-time entries to a French blog, contribute to understanding a unique, collective expression of men’s lived experiences of prostate cancer. These anonymous survivors shared their varied reactions, ways of coping, and thoughts on needed change.

## Background

Prostate cancer (PC) experiences usually embody multidimensional issues of quality of life (QOL) for men and their families [1–7]. Men’s subjective experiences of PC involve the construction of a social representation of PC that is bound by a specific time and culture [8]. By ‘social representation’, we mean a way of knowing and thinking socially, that generates a common sense meaning of one’s life [9]. Social representations are grounded in cultural values, and depend on the social reality individuals face daily [10]. In France, a public debate involving health and communication professionals and social analysts as well as lay individuals and group of survivors has been ongoing since 2008 when the Haute Autorité de Santé recommended against systematic screening for PC using the prostate-specific antigen test (PSA) among men aged 50 years and older [11]. The subsequent polemics and uncertainties expressed by health care providers and the public has affected men’s level of prostate cancer literacy. Public health policies have the potential to impact social determinants of health, and together, they affect the level of health literacy (HL) and, consequently, quality of life [12].

As HL embodies a set of skills and abilities related to the identification of a credible source of health information, the coding and interpretation of information until its application in a safe way to self-care and self-manage needs and situations provoked by the natural trajectory of a disease [13]. Consequently, becoming health literate implies favourable conditions to live under better life conditions in consonance with changes and limitations provoked by degenerative disease like cancer, particularly PC [14].We analyzed the testimonies of PC patients and survivors posted to a public blog hosted by a French national prostate cancer patients’ association; this was done as part of the fieldwork for a critical ethnographic study [15] (Zanchetta et al., *PC representation among Francophile/Francophone men -* unpublished manuscript) of the expressed values, behaviours, beliefs and language of Francophone and Francophile men experiencing PC, as well as its related representations. The aforementioned study was framed by social representation theory, specifically in terms of the *core* (versus *peripheral*) elements of certain beliefs [10, 16]. The core of a representation is stable and regardless of new experiences, ideas or knowledge it remains unchangeable. This core can generate or change the meaning and the value of a given social representation. The core organizes the links between peripheral elements of the social representation. Depending on one’s interpretation of the outcome of new experiences and learning within the social world, these peripheral elements can be modified. In other words, the elements comprising a social representation depend also on the interface between its core and the reality out of which the social representation comes as modifiable elements, the peripheral ones [10].

We hypothesized that these testimonies would reveal the core social representation of men’s experiences of PC, and that the bloggers’ predominant views and perceptions of PC as expressed in their discourse would reflect a core representation of PC, grounded in their lived experiences. We were especially interested in the bloggers’ overall perceptions of PC as it affected their subjective quality of life.

### Patients’ subjective quality of life

According to the philosophy of client/patient-centered care [17], patients’ social worlds and subjective experiences are relevant for understanding their engagement in health care. Certain social determinants of health, such as social support networks, education and literacy, personal health practices and coping skills, gender, access to health services, and culture [18], are known to influence patients’ engagement in health care decision-making. Examples of such influence include how patients embrace the popular culture regarding their illnesses, and the social representations of certain diseases. Prostate cancer is a disease with strong social meaning in that it threatens male identity, and is linked to stigma and vulnerability [3–5] due to the potential loss in sexual potency, libido, functional erection and ejaculation [8]. These associations can interfere with men’s motivation in PC self-management, a critical clinical issue that is not sufficiently understood and documented [19]. Prostate cancer discourse and the information that patients share amongst themselves are important sources of understanding for health care professionals (HCPs). Professionals should identify and appreciate cancer patients’ experiences [20] as disclosed in illness blogs [21], including patients’ preferences in their plans of care [21, 22], daily difficulties in self-care, and the type of information they need to adapt to life with cancer. Such sources can also provide insight into the value of patients’ know-how to create innovative (if perhaps unconventional) self-care and self-management strategies, and how those strategies work (or do not). Ultimately, PC patient discourse can reveal obstacles or facilitators to patients’ engagement, men’s satisfaction with the health care system and its effectiveness as a support during their PC trajectory, and critical feedback about what health information is needed and can improve patient QOL.

Quantitative and qualitative studies have demonstrated that QOL among patients with PC is influenced by men’s physical, psychological, and social role functioning, as well as the disease or treatment-related symptoms they experience during the pre-treatment, treatment and post-treatment phases [1, 2]. The lack or severity of symptoms experienced prior to diagnosis and the pre-treatment phase influences physical functioning [1, 2]. The quality of communication at the time of diagnosis and rigid views of masculinity seem to affect men’s perception of their psychological functioning [2]. Availability of information on treatment options can either empower men in their decision-making, or overwhelm them so that they relinquish decision-making to their HCPs [6, 23]. Psychological aspects include the pressure and stress of uncertainty prior to decision-making, usually followed by relief [1, 6, 24]; beyond this, feelings of tension, worry, irritability, fears of relapse and death can be mixed with an increased sense of gratitude and appreciation for life [1].

Called a “relationship disease” [25, 26] due to its impact on personal relationships and the difficult emotions involved, PC affects all men regardless of their sexual orientation or the stability of their marital relationships [1, 27]. A notable impact on their social role functioning is men’s unwillingness or hesitancy to bring up PC with friends and relatives; this has negative repercussions on their physical/psychological functioning by leaving men physically tired and emotionally exhausted from trying to maintain a stoic appearance [1, 3, 5–8, 19]. For instance, voluntary home confinement may occur to avoid having to manage urinary incontinence in public settings [28]; this is also associated with an altered sense of one’s masculinity, typically viewed as being feminized and infantilized [1, 28].

A passive tolerance of symptoms [29, 30] from treatment-related side-effects can affect QOL. However, as the idea of survival prevails and less emphasis over time is placed on symptoms, some limited lifestyle adaptation occurs [27, 30]. The major QOL issue is the loss of sexual function from reduced penile length and erectile dysfunction [3–7]. Despite remediation with medications, altered sexual practices and use of erectile aids are not easily accepted by heterosexual men [31].

### Importance of studying blogs and blogging

Blogs initiated by health organizations, corporations, professional associations and lay groups are considered to be “the most old, most established, and evaluated form of social media” [32]. Chung and Kim’s [33] seminal work on cancer patients’ and their significant ones’ use of blogs launched multiple studies on the benefits of blogging to patients, HCPs and researchers. Patients who participate in a digital social network benefit in an impressively wide range of ways, including (a) support that contributes to sound decision-making [33–38]; (b) better emotion management, connectedness and support, increased problem-solving skills, and opportunities for information sharing [20, 21, 33, 36–42]; (c) taking advantage of the collective wisdom of other bloggers and HCPs [43]; (d) feeling empowered to take more responsibility for participating in clinical, financial and health-related decisions [33]; (e) having an advocacy platform for patients and their families [20, 21, 32, 37, 41], whose influence on policy is extended by media coverage [32, 44]; and (f) the privacy and freedom of anonymous blogging [36, 45, 46] which can facilitate a sense of social identity and belonging [47] while avoiding social stigma [48] and aiding the search for information [49].

For HCPs, blogs foster open access to and exchange of information with the lay public; blogs can foster a communal, collaborative dialogue with patients and their families [33, 39]. Blogs can facilitate HCPs’ understanding of patients’ illness experience [20], perceived QOL [21] and preferences, all of which provides crucial evidence for the improvement of heath care services [50]. Since digital inclusion is fundamental to achieving these benefits equitably, the removal of barriers to computer and Internet access is critical [39, 51]. Currently, those with high levels of education are more likely to use the Internet to seek health information [52], so efforts to improve access to e-health services, including blogs on cancer management, are warranted [53]. Feedback from patients’ postings can also be used by health care managers and policy makers to improve health care services and policies [20, 33, 35, 39, 41, 54].

Blogs are a new topic of health research, but since around 2008 [35, 45] studies have explored how different types of blogs are used by the public for health-related information and purposes [35, 41, 45]. The exchange of information between patients on blogs remains understudied [20, 39, 42, 55], with few studies addressing the actual impact of blogging on health management behaviour [35, 40, 56] and related decision-making [38]. Further research is warranted on patients’ use of blogs for health-related information and experiences [57], and it has the advantage of being a fast, low-cost, and informative way of obtaining qualitative data, even internationally [55]. For instance, the launching of medical blogs in the USA has increased exponentially [44], and blog use increases yearly [42], making blogs an interesting source of input from hard-to-reach and global audiences [33, 35, 36, 39, 40, 44, 45, 58], and particularly relevant for information on health promotion, self-management and self-care.

Studies have rarely addressed how to tailor information dissemination for a particular patient group [38]. Since most research on blogs focuses on individual bloggers, few studies have reported on cancer patient-led online communities [20], where patients create and participate in blogs as both authors and readers, and comment on the impact of their illness [42, 59]. The current study of a PC patient-led blog aims to explore the illness experience, and thereby address this knowledge gap, identified by Kim and Lee [42].

We chose to study the open-access, PC survivors-led blog hosted by the French National Association of Prostate Cancer Patients (Association nationale des malades du cancer de la prostate-ANAMACAP) due to the host’s credibility and reputation as an entity that supports and advocates for survivors and their significant ones. ANAMACAP is well-known among cancer organizations, health care organizations and HCPs, who frequently recommend that their clients contact ANAMACAP to obtain peer support and information as well as establish social networking. The ANAMACAP blog provides answers written by HCPs to bloggers’ questions and posted comments (a feature not accessed by the general public). This site is also widely used by patients and their significant ones to exchange information, and is monitored by HCPs. Thus concerns about the quality of PC-related information available to information seekers (and the testimonies analyzed for this study) are mitigated.

Blogging has become an avenue for PC discourse, which can provide further insight into patients’ illness trajectories in order to identify patient needs and improve health care services. New understandings of PC patient issues and QOL may be revealed due to the privacy afforded by anonymous commentary on sensitive information [36, 44, 46] which would otherwise be less likely to be expressed given the stigmatized nature of PC [48].

This study aimed to explore issues of QOL as reported by French PC survivors in a public blog, and had two objectives: (a) to identify the salient aspects and issues of the experience of living with PC from the perspective of PC survivors based on textual data from their posted testimonies; and (b) to analyze the ideas in the posted testimonies about perceived and lived impacts of PC on QOL.

### Conceptual framework

Quality of life is a multidimensional and complex construct and due to its subjective nature, a consensual theoretical definition has not yet been established [60]. Nevertheless, we adopted Haberman and Bush’s conceptual framework which presents QOL in four basic dimensions: (a) physical functioning, (b) psychological functioning, (c) social role functioning, and (d) disease- or treatment-related symptoms. [60] To elaborate: physical functioning relates to physiological changes affecting activities of daily living, which may compromise one’s ability to perform work, sports, leisure activities, self-care, and may influence one’s diet, and hygiene management. Psychological functioning includes one’s mood state, cognitive ability, perceptions of well-being, transcendent behaviours, and attitudes towards self-body image, self-esteem, and self-efficacy. Social role functioning involves relationships in one’s personal life, at work, with peers, and in society at large. Disease- or treatment-related symptoms refer to physical side effects related to the disease process, and are often seen as a consequence of therapy [60]. We applied this framework of QOL components and indicators to analyse a set of testimonies posted to a PC survivors’ blog.

### Guiding exploratory question

What aspects of French bloggers’ testimonies about their lived experiences with PC indicate the predominant features of QOL in community settings and underpin their collective social representation of PC?

## Method

### Design and procedure

This content analysis of blog data was conducted as part of a larger ethnographic study [15] designed to explore the social representation of PC among patients and survivors living in France (both French-born and immigrants to France). An ethnographic design allows for the description and interpretation of the shared and learned patterns of values, behaviours, beliefs and language of a cultural group or community. A critical ethnography approach [15] was used to guide the fieldwork whose objective was to understand the social context and predominant issues affecting men’s varied experiences of PC and how they are affected by differences in knowledge, power and social position.

The study was conducted within the “context of living” [61] (p. 19); this allowed the principal investigator to understand the social context of PC survivors. The fieldwork included a 4-month period of immersion in the French PC culture through interviews and discussion with health and social sciences professionals, being exposed to popular media regarding PC, touring hospitals and discussing PC patient care, and speaking to patients and survivors in various stages of treatment, recovery and rehabilitation. From all this, a particular understanding emerged of the health and social context of having PC in France.

The anonymity of the blog postings is a methodological asset in obtaining survivors’ authentic comments, including the reporting of sensitive information and adverse or negative events [36, 45, 46] blogging can be a coping strategy, a form of self-therapy, and a way to receive empathy and support from other bloggers [33, 39, 41, 42, 56, 62]. Collecting data from a public PC survivors’ blog is an invaluable strategy for revealing critical underlying issues while filling in psycho-oncology knowledge gaps about cancer cultures and patients’ view of cancer – for instance, as a social plague [63]. This data source reveals an implicit medical discourse (expressed by HCPs), along with the lived experiences grounded in survivors’ accounts, as well as a popular version of scientific representations of PC within French culture, which altogether configures a particular transcultural perspective.

To accomplish this, we applied a media content analysis method [64, 65] to the textual data drawn from the blog testimonies. This method explores an idea or a recurrent theme within analytical units in different types of documents/artefacts. In this study, each posted testimony was considered an analytical unit. Usually, a general, simple analytical question guides the retrieval of content from the units to detect patterns of what is similar and repeated. We analyzed the data to explore the general tone of the discourse, the period of document production, the documents’ authors, types of topics, the social norms and behavioural codes, public awareness of the subject matter, and the social interactions among individuals.

This analytical method allowed us to identify embedded ideas about PC, mainly by the tone of messages posted by bloggers. The cultural portrait of bloggers as a survivor social group emerged in terms of their social identity, codes of virtual social solidarity, scientific awareness and literacy, and comments regarding autonomy and self-management, as well as awareness of inequities and power imbalance when dealing with the health care system. Together, these features revealed the scope of the systemic challenges bloggers faced in relation to their QOL (Table 1).

**Table 1:** Overview of the Analytical Process

Steps	Procedures
Step 1- Create a retrieval table using Excel software to display entries of blogger testimony	Define 16 general categories of empirical data: chronological ID number, blogger’s fictitious name, sex, age, PC stage, exams undergone, comments about PC-related exams, treatment undergone, comments about PC-related treatments, experienced complications, comments about PC-related complications, message topic, message tone, message goal, and number of posts; Populating the table with retrieved data with distribution of categories by rows
Step 2- Retrieve testimonial content	Critical reading to retrieve content relevant to research questions, select and compile content as incidents in the retrieval table cross-referenced by the categories listed above, count all entries per category, interpret content in table vertically and horizontally.
Step 3- Re-order the categories of empirical evidence	Rank data by frequencies from the highest to the lowest incidence.
Step 4- Conceptual reduction	Rearrange the 16 categories of data to 5 key topics and 13 key features (see Table 2), reduce 16 general categories to 7 major categories and 25 sub-items, identify testimonies per sub-items and their respective frequency, and create a summary table (see Table 3)
Step 5-Conceptual correspondence	Attribute categories of data according to their fit with the four basic dimensions of the QOL conceptual framework.
Step 6-Verbatim search	Identify quotes that are representative of each of the four basic dimensions of the QOL conceptual framework.

The retrieval period was determined after initial contact with the field and was cued by the aforementioned health authority decision to recommend against prostate specific antigen (PSA) systematic testing for PC screening, an announcement that provoked major media attention and public debate in France. Thus, purposeful sampling [66] was framed by survivors’ reactions to the health authority decision. The first and third authors, both of whom are bilingual (English/French), conducted the first critical reading of all the testimonies, identifying content that addressed the four topics of interest identified in the blog testimonies: medical care and rehabilitation; recovery post-PC; health education and self-care; and global vision of PC and its impacts on the sense of being a man. The type of language used in the messages was also assessed (Table 2). No coding process (manual or software aided) was used since the media content analysis method does not require it but several descriptors were ‘counted’, such as the number of posts that used a ‘cautious tone’, or complained about poor medical practices. The blog data was categorized according to pre-established recurrent themes. Frequencies were calculated using Excel Office 2011.

**Table 2:** Scope of Retrieval from Blog Testimonies

Subject	Specific areas
Medical care and rehabilitation	Treatment methods and their impact on the life of the patient and his family Importance of patients’ involvement and participation in the treatment and rehabilitation trajectory Self-care needs during treatment and rehabilitation period
Recovery post-PC treatment	Consequences on daily life during post-treatment recovery period, specific to: - work life - sexual functioning - physical energy level - psychological wellbeing - broad social life Self-care needs during recovery time Possibility of side effects and how to face them
Health education and self-care	Availability of formal information sources Availability of informal information sources Highlights of support groups for men and their families Hidden discourse and silence between men with regards to PC Women’s roles during each stage of PC trajectory
Global vision of PC and its impacts on one’s sense of being a man	Impact of changes and dysfunctions on: - the ability to make autonomous decisions - physical autonomy for self-care - changes in physical functioning (micturition, excretion, and ejaculation) - sexual capabilities - erection - libido - emotional control - masculine self-image - image as family bread-winner - awareness of end-of-life - self-realisation
Type of language used in the messages	Message tone: - informative - alarmist - sensationalist - educative - critique - political

The following quotes extracted from the blog testimonies were translated from French to English by the third author and verified by the first author.

**Fig. 1:**
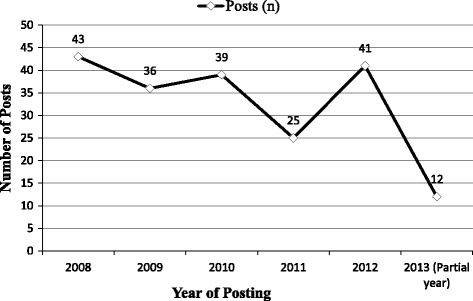
Distribution of posts by year (n=196)

The blog entries (*n* = 196) selected for analysis were retrieved on July 17th, 2013, and included posts made over several years, between January 2008 and June 2013. This time frame matched a period during which media attention was high and public discussion evident in response to the Haute Autorité de Santé’s decision on PC screening. The distribution of posts by year is presented in Fig. 1.

To ensure methodological rigour in the analytical work, we applied verification procedures for qualitative research as an *incitement to discourse* [15]. Specifically, we triangulated the data analysis and confirmed the results by testing the viability of patterns, getting comments from experts and informants, and auditing by external examiners [67]. The quest for interpretative credibility aimed to respond to different audiences about issues of culture, ideology, gender, textual language, relevance, advocacy, and standards of respectability [15].

In June 2013, the first and second authors reviewed the raw material and verified its appropriateness and clarity as empirical data that could reveal various aspects of men’s experience of PC within the French health care system. The same authors interviewed HCPs (a physician and two psychologists) at the League française contre le cancer, who broadened their understanding of the broader context or macro-perspective of living with PC in France, referring to the influence of social, cultural and political factors throughout the PC clinical trajectory, from screening to rehabilitation. Multiple conversations between the first author and a League volunteer and PC survivor were beneficial for understanding his unique perspective as a natural knower. The knowledge gained from these discussions assisted the researchers with preliminary data interpretation, specifically by providing a context for how the blog data could reflect men’s lived indicators of QOL.

In May 2014, the preliminary data analysis was discussed with a researcher located at Paris (the second author), who corroborated the Toronto team’s (the first, third and fourth authors) interpretations of the findings. From November 2014 to April 2015, the Canadian research team completed the final analysis by applying the health-related QOL conceptual framework and its four dimensions. The final data interpretation was confirmed by a Canadian advanced practice nurse who has extensive clinical experience with PC patients. Moreover, the review of an early draft of this manuscript by two other Canadian nurse researchers in the area of PC and chronic illness/QOL completed the procedures of verification.

For the analysis, the testimonies were arranged and classified by the following categories: diagnostic exams, type of treatment method, secondary effects/complications, message topic, message tone, message objective, and posting frequency. The categories relating to the physical and medical trajectory of PC were further subdivided into aspects of personal lived experience or content based on knowledge of others’ experiences, or from general knowledge (Table 3).

**Table 3:** Summary of the Most Salient Indicators from Blog Testimonies (*n* = 196)

Categories	Testimonies (n)	Percentage (%)
Diagnostic exams		
PSA testing	120	61%
Biopsy	73	37%
Type of treatment method		
Prostatectomy	101	52%
Medication class:		
Prostaglandin	39	20%
Hormonal anti-neoplastic drugs	36	18%
Impotence drugs	21	11%
Hormone therapy	27	14%
Radiotherapy	25	13%
Secondary effects/complication		
Erectile dysfunctions	83	42%
Urinary incontinence	74	38%
Anxiety/Fear	45	23%
Pain	36	18%
Message topic		
Encouragement	65	33%
Informative-resources	54	28%
Seeking information	41	21%
Complaint	31	16%
Denunciation	23	12%
Message tone		
Positive	95	48%
Neutral	52	27%
Negative	40	20%
Political	12	6%
Message objective		
Share one’s experience	179	91%
Provide information	51	26%
Teaching about PC	9	5%
Posting frequency		
Once	151	77%
Twice	21	11%
Three times^a^	9	5%

^a^Note: The sum of posting frequencies does not equal to *n* = 196 because testimonial content display ideas that were classified in multiple indicators

## Results

The results were organized by three categories: the bloggers’ self-identification, reactions to experiences, and impacts on QOL. References to social determinants of health tended to be embedded in the posted testimonies. For ethical reasons, the bloggers’ fictitious screen-names were omitted from the quotes below.

### Self-identification

The bloggers usually identified themselves by their diagnosis, results, treatment method, and rehabilitation; only very few bloggers mentioned their social identities, whether as husbands (*n* = 30; 15%), fathers (*n* = 6; 3%), or professionals (*n* = 4; 2%). Their ‘patient’ identity or health status was described primarily in terms of medical metrics, which is, their Gleason score and prostate specific antigen (PSA) titration. By doing so, bloggers portrayed features of a new social identity as members of a sub-culture of cancer survivors. More specifically, 61% (*n* = 120) of the bloggers started their narrative with information about PSA results, and 26% (*n* = 51) others cited their Gleason scores, as the following example shows:

*Biopsy and PSA of 10.5. Positive results GLEASON 3 + 2.… Clinical results: PSA was at 1.5 mg/mL, 3 months after the intervention, 0.9 three months later, then 0.5, fluctuating around this value until March 2004*.

Other forms of identification (see Table 3) included treatment methods such as prostatectomy (*n* = 101; 52%), biopsies (*n* = 73; 37%), hormone therapy (*n* = 27; 14%), and radiotherapy (*n* = 25; 13%). Experiences of secondary effects/complications were also cited, such as erectile dysfunction (*n* = 83; 42%), urinary incontinence (*n* = 74; 38%), and psychological distress such as anxiety or fear (*n* = 45; 23%) and pain (*n* = 36; 18%). Similarities in their shared experiences helped to define a particular sense of belonging.

*I wish to share an experience that many have gone through before me and, if necessary, collect advice from my “brothers in disgrace”…*

### Reactions to experiences

The bloggers demanded and expressed their desire for specific forms of treatment, and encouraged certain actions, including their right to choose their physician. Views on access to appropriate and safe health care were discussed, sometimes with fervour. They forewarned each other about future challenges. They used the blog to alert other men, to urge their Association’s president to take stronger political action, and to search for solutions.

*My advice: start monitoring and interpret the numbers properly; be proactive instead of reacting with resignation, just subjecting yourself to the doctors’ more or less adequate actions…*

As for dealing with negative experiences, bloggers commented on their use of coping and problem-solving skills, and freely disseminated information and suggestions on how to avoid specific problems. Most of the testimonies concerned developing a supportive, accepting environment. They expressed a sense of brotherhood, stoicism, and humour, mainly related to loss of their sexual potency; some bloggers disclosed their use of penile injections (*n* = 39; 20%) and urinary catheters (*n* = 32; 16%), sometimes in terms that suggested those procedures violated their physical integrity.

The bloggers shared their own experiences (*n* = 179; 91%) and used a cautious tone, sometimes to encourage others (*n* = 65; 33%), and other times to seek information (*n* = 41; 21%). The blog was occasionally used to denounce medical misconduct and poor practices of HCPs (*n* = 23; 12%), citing poor health outcomes and unpleasant attitudes during care. One blogger, who was a physician as well as a PC survivor, expressed clear resentment over the unsympathetic manner of other physicians:

*As a doctor [and patient, I was] … “let down” by my fellow doctors.… Thanks to my dear colleagues, who never bothered treating me as a human being … but dealt with me according to general protocols….*

The large majority of testimonies (*n* = 95; 48%) maintained a positive tone in their messages regarding decision-making attitudes as illustrated by a man’s concluding thoughts:

*Stay calm and don’t panic because the medical field has mastered well enough this widespread disease; carefully select your hospital institution…*

Some testimonies were also in a neutral tone (*n* = 52; 27%), with no judgment of values, expression of feelings, nor appraisal of the situation :

*I then decided to take a step back, to inform myself by whatever means possible, and to consult other specialists before doing anything.*

In comparison, testimonies reporting negative tones (*n* = 40; 20%) were the least common and are exemplified below:

*I was seen by this Professor who proposed no other alternative.… The dialogue turned out to be difficult.… I won’t mention the outrageous incompetence … bordering on verbal abuse. … I’ll never consult this «specialist» again…*

It is notable that bloggers generally neglected the opportunity to engage or build an online community for learning, advocacy and/or mutual help (see Table 2); the frequency of one-time postings was high (*n* = 151; 77%). One exceptional blogger posted 14 times, providing updates on his disease trajectory while strongly advocating for his chosen treatment option. When a blogger responded (*n* = 26; 13% of posts) to another’s previous post, the exchange was clearly intended to provide emotional or informational support, but quickly died out within two (*n* = 21; 11%) to three (*n* = 9; 5%) replies. While the blog provided anonymity, it equally prevented the reader from forming any sort of personal connection with other faceless bloggers.

### Impacts on quality of life

The data illustrated how PC affected men’s functioning. We distinguished these data according to the four QOL framework dimensions, although the content was frequently mixed.

### Physical functioning

Some bloggers were able to fully resume their previous lifestyle (*n* = 47; 24%) after the treatment was done and their recovery was complete. They returned to the same level of activities (social, sports, and work), sometimes with minor limitations (*n* = 53; 27%), and in some cases, even adopted better lifestyle practices (*n* = 8; 4%).

*Next week I’m taking up sport again.… Since my operation, I walk twice a day (for half an hour) [along with] my other activities.… Life continues, and it’s wonderful!!*

In other instances, diagnosis and/or treatment of PC motivated bloggers to make changes to their lifestyle in terms of nutrition and physical activity as a preventative measure (particularly in conjunction with active surveillance of PC), to slow down PC progression and decrease the odds of recurrence.

*I drastically changed my nutrition by eliminating all products containing gluten, dairy products, and decreasing [the amount of] added sugar.…*

A few bloggers (*n* = 6; 3%) addressed the impact of PC on their readiness to resume their professional activities, sometimes in a non-supportive work environment. One blogger noted that going to work was a coping strategy for him to deal with low morale: “My mood isn’t good but I continue to work because it seems like it’s helping me to cope.” For others, the required recovery time away from work was not a welcome prospect, possibly for financial reasons. The side effects of treatment were extensive and lasted for an extended period of time, consequently affecting their activities of daily life (*n* = 53; 27%), psychological state (*n* = 26; 13%), marital relations (*n* = 9; 5%), and/or their ability to resume work (*n* = 1; 1%).

### Psychological and social role functioning

Posts that mentioned psychological effects and social role functioning were frequently intertwined with other aspects, making it unfeasible to differentiate between them. Bloggers reported feeling surprised or shocked upon hearing of the possibility of having PC and when receiving a confirmatory diagnosis of PC. They expressed fears in relation to having PC and deciding which treatment method to choose and the possible consequences; body image and self-esteem were affected when side effects like urinary incontinence, erectile dysfunction, or pain arose, sometimes leading to the onset of depression. Despite this, bloggers expressed a desire to remain in control, as evidenced by their attitudes of self-efficacy and determination to learn about PC, seek advice from others or Internet resources, and personally choose their treatment method. Keeping a positive attitude and outlook on life helped with the lived experience of PC. Only three bloggers (2%) were confident about being in remission, regardless of how long treatment and recovery took, and maintained a positive outlook on life.

*… To date no leaking and perfect libido. I resumed walking and biking.*

While references to marital partners were infrequent, a few bloggers (*n* = 11; 6%) referred to the physical and emotional support received from their spouse as an effective coping strategy, helping them through their ordeal. They mentioned the experience of living with PC as a couple (*n* = 6; 3%), thereby strengthening their bond and reinforcing their commitment towards one another. Four bloggers (2%) transcended physical sexual activity for other forms of conveying their feelings to their partners through communication, gentle caress, and companionship. For others, PC undermined their marital relationships (*n* = 9; 5%) due to compromised sexual life after treatment. This impairment contributed to the decision of two bloggers (1%) to seek a divorce, and adopt a positive attitude to seeking new relationships.

Relationships with HCPs were mentioned frequently, and bloggers stressed the importance of carefully choosing HCPs. Those who had a quick recovery and only minor side effects were more likely to praise and attribute their successful recovery to their health care team. For some, the decision of which treatment method to choose was influenced by information provided by their physician, the manner in which this information was conveyed, and the health care partnership throughout PC treatment. Sixteen bloggers (8%) followed their physician’s recommendations without question, while others (*n* = 20; 10%) advocated informing oneself from various sources and seeking advice from others, but taking control and ownership of their decisions.

The blog was also used as a channel for political action, to advocate for better access to information, medication, use of sexual devices, reimbursement of expenses, and even to urge changes in health practices.

*Like many of us, I’m thinking of getting a vacuum pump and I find it unfortunate that we cannot try one beforehand, like Mrs. X suggested at Z Hospital. This service exists in the US, then why not in France?*

*Like a lot of you, I think that improvements could be made in the promotion of this equipment that provides us much appreciated help.*

### PC-treatment-related issues

Posts that referred to medications and treatment-related consequences cited 44 brands of medications falling in 14 different drug classes, including those to treat erectile dysfunction by prostaglandin penile injections (*n* = 39; 20%) or impotence drugs (*n* = 21; 11%), and hormonal anti-neoplastic drugs (*n* = 36; 18%) to treat advanced PC (see Table 3). The posts mentioned adverse effects of medications for erectile dysfunction, including headaches, heart rate alterations, severe hypotension, and prolonged erections. Hormonal anti-neoplastic drugs disrupted hormonal balance, which in turn resulted in sweating, hot flashes, and gynecomastia [68, 69].

Urinary incontinence, a major treatment consequence, was commonly addressed by pre- and post-surgery perineal reinforcement exercises. However, three bloggers (2%) revealed that they adopted penile clips and liners or taped their penis to prevent leakage and more comfortably engage in work, leisure, or sport activities.

To manage their erectile dysfunction, some bloggers recommended penile injections (*n* = 39, 20%) and vacuum pumps (*n* = 10; 5%), and even cited websites for purchasing equipment and instructions:

*I prepared this method … from my extensive internet search … price of a high-end vacuum: http://… Videos regarding using the pump for erections: http://…*

Instructional content was not common, but one post from a man who biked was pedagogically interesting. He explained in detail his simple, efficient procedure for dealing with urinary incontinence:

*It’s a simple system: to close the urethra, I use a short rope (60 cm) of large diameter and soft to the touch (very important: so as not to hurt the foreskin); this type of rope can easily be found in supermarkets. With this rope, I make a clove hitch knot around the shaft, being very careful to place it above the glans, the foreskin, without tightening too much. The clove hitch knot, consisting of two interconnected loops, seems to me like a good solution: it is easy to do and undo; furthermore, the double loops extend the clamping surface and will not hurt if done properly. Then I extend this short rope by another longer one, so as to go around my waist: in this manner, the shaft remains upright and pressed against the lower abdomen.*

Overall, the men’s testimonies portrayed PC as an isolated and isolating experience, a mostly solitary burden. The few responses (*n* = 26; 13%) made to previous postings may be understood as an attempt from the responder to provide mutual support, encouragement, education, and guidance. It could be interpreted as a reply to the perceived burden expressed in the posting. Personal engagement and promoting a critical attitude towards decision-making and health consumers’ rights seemed to be the main functions of the blog. In reaction to the effects of temporary and permanent dysfunctions that impaired their QOL, the bloggers expressed feelings of resignation, attitudes of acceptance, and demonstrated how creative skills were used to successfully deal with their PC-related issues.

Overcoming all forms of isolation would be particularly challenging to survivors possessing low literacy level or a low proficiency in French language. At risk of reinforcing their social exclusion, subtle request for sophisticated writing skills such as “your question deserves to be in this site. Please reformulate your question in good French…” would undermine the collective work to promote digital inclusion for cancer patients [45].

## Discussion

How men interacted within the blog community reflects a desire to provide mutual help and a social support network. The testimonies exemplified the use of a variety of coping strategies, personal health practices and self-management of PC. Posts regarding both successful and failed self-care strategies were openly disclosed. This suggests that bloggers shared unique and valuable information for coping with daily PC-related issues, information that may be a useful complement to HCPs’ expertise, something that clinicians may not necessarily provide [70]. As knowledgeable experts, patients can testify about the safety and efficacy of their self-care techniques and self-management of PC post-treatment consequences. It is noteworthy to recall that from the perspective of a transdisciplinary model of evidence-based practice, not enough attention and importance has been given to patients’ preferences, actions, values, needs, and experiential knowledge [54]. In other words, scientific evidence is seen as “a necessary but not a sufficient aspect of clinical decision-making” [54] (p. 373). The context of e-health is supporting the emergence of patients’ expertise, skills, and resilience, as well as expression of their needs and priorities; it allows for full participation in decision-making in their new role as “e-patients (empowerment, engaged, equipped, enabled)” [50] (p. 148) and can result in the adoption of behaviors leading to autonomy, emancipation, and self-determination, as well as the transfer of self-care skills to other groups of patients [70].

Another important aspect of this blog culture, related to social support, is the expression of a masculinity framed in terms of brotherhood. The men’s advice and warnings suggest ways to navigate the health care system in order to have access to better services. The anonymous nature of blog posting apparently made it easier for men to share their subjective experience openly, including emotions, affect, and attitudes [71]. These types of disclosures contrast with the mainly functional representation of PC revealed in an interview-based study with Anglophone Canadian men of European descent [8]. In those interviews PC was perceived as a simple, uncontrolled biological process leading to damaging consequences and painful feelings. Experiences of loss in sexual desire and potency, functional erection and ejaculation, and damaging consequences to sexual life, requiring lifestyle changes, especially due to urinary incontinence, co-existed with a hopeful, positive vision of life. In yet another study, Franco-Canadian men who were interviewed also portrayed PC as a relatively innocuous disease and discussed the biometrics in primarily bio-technical or functional terms, with a focus on level of safety and treatment effectiveness [72].

The results of this study indicated that the content of the bloggers’ posts oscillated between a fatalistic view of a ‘brotherhood of disgrace’ and a social advocacy perspective whereby men exercised their voice to warn peers about pitfalls while supporting them by sharing experiential knowledge to promote well-being for all [23, 24]. An assumed sense of unity led to a sharing of experience, information, and emotional support [73], including expressions of gratitude for information on self-care. The results corroborated findings from other studies of blogs in that the act of writing in a blog revealed engagement in self-care [33] and more opportunities to gather information [20, 21, 33, 36–42].

The blog provides a *post-facto* opportunity to congregate and seek social solidarity to overcome transitory and long-lasting dysfunctions. The physical impacts of PC affect more than men’s social and interpersonal relationships; they challenge the dimension of spiritual well-being and emotional safety [33, 71, 74]. QOL can be gravely affected by the severity of these complications, along with men’s ability to devise self-care and self-management strategies to cope with them. Interestingly, the blog testimonies in this study only infrequently referred to support from significant others, which previous studies have shown to be helpful to enhance men’s self-care and assist them to resume their usual activities of work, leisure, or sports, and therefore resume their social life [33, 71, 74].

The language used in the testimonies revealed that political, educative and compassionate discourses were intertwined to create a complex representation of PC. The anonymity of using the blog allowed men to disclose failures, make denunciations, freely address morally challenging issues, and discuss self-care strategies that may be less socially acceptable to speak of in person [23, 71]. The blog offered a public space that was used to teach, learn, advocate, and complain about aspects of their experience of PC, to reveal a subjective perception of it as a disease that reinforces a traditional paradigm of masculinity [24, 74], one that can act as a protective buffer, or as a risk factor for disease prevention and effective self-management [75]. Paradoxically, the blog may serve as a mechanism for challenging the dominant paradigm of traditional masculinity and, at the same time, reinforce the invisibility or silencing of men’s full range of feelings outside the blog [71]. According to the predominant norms, men are expected to be assertive or aggressive, seek immediate solutions, maintain strong social bonds, and apply rational decision-making skills within a culture of assumed invulnerability, toughness, autonomy, resignation, and stoicism [76–78].

The blog entries on social encounters with physicians reflected a predominantly paternalist style of interaction. Patients’ trusting acceptance of professional authority and expertise was also linked to the acknowledgement of mistakes and misleading behaviours [23, 71]. Indeed, those who trusted their health care providers were less likely to seek secondary opinions [77]. While paternalism can protect patients from invalid sources of information, such a disciplinary style also inhibits men from seeking information from other sources and can undermine successful self-management strategies [77, 78]. The testimonies revealed a sense of regret over not having previously sought any PC-related information and other preventive health care resources available to the general public, as this could have helped with earlier detection, improved their PC literacy, and the potential prevention or reduction of adverse effects [77].

With increasing Internet use and easy access to all types of health information of varying degrees of accuracy, policy-makers face the challenge of making sure truthful PC-related information is available to those seeking to educate themselves, and redirecting individuals to the availability of these resources. No consensus exists on whether it is appropriate to incorporate patient-led blogs as a new source of healthcare information. As an innovation in health care organizations, patients’ blog content and exchange of experiential knowledge may be worrisome to health care managers, and such blogs may face significant structural, cultural and financial barriers [77, 79]. Moreover, such patient blogs prompt the need to define standards of quality, safety, accountability and responsiveness, plus a system for knowledge management [58].

Various suggestions have been offered to address these concerns. A blog collaboration between patient groups and HCPs could make e-health information understandable, with trustworthy content tailored to various user groups and made available on an easy-to-use e-platform. [53] Other strategies to increase the reliability and quality of e-health information [41, 42, 44] include the explicit citing of sources and the review and confirmation of content by HCPs who could also correct errors and misconceptions and provide additional information/answers to bloggers’ questions [35, 39, 44] and hyperlinks [33]. Such controls could increase a blog’s perceived credibility, as well as clarify the HCPs’ role in endorsing, promoting and facilitating the use of blogs for health-related purposes [53].

Scientific evidence on the benefits of blogging to both patients and HCPs points to the need to redesign professional roles to accommodate the inherent demands of this innovation. For instance, an advanced practice nurse as knowledge broker could support an organization-based website with health information based on evidence-based practice and new institutional policies [80], which incorporate a blogging feature as a strategy to follow-up patients’ progress in safe self-management. Another example regards oncology nurses involved in the implementation of a rapid cancer diagnosis system who are called to respond to the demands of information dynamics by working with IT staff to create a common e-health platform that provides an interactive source of information suitable for professionals and clientele [81]. In fact, emerging evidence suggests the sort of features required to design a safe and high quality system of health blogging that can contribute to a knowledge alliance. As a result, we can foresee progress in patient safety and autonomy and empowerment based on medical knowledge and new avenues of communication. One example is the Mayo Clinic (USA), which is currently considered to be the gold standard in terms of blogs’ usefulness for both patients and providers, as well as the quality of information it contains [57].

At this stage of development, this sort of innovation presents challenges for evaluation and the design of evaluative studies, since there is no consensus on methodological and ethical procedures. Researchers studying blogs and blogging should remain cautious in dealing with issues of authenticity and sampling [32, 55, 58], as well as of informed consent (particularly, privacy) for data collection and use [41, 55].

## Conclusion

As social media is becoming a common source of health information (although it may not be the best platform for peer-to-peer social support), HCPs need to be critically aware of social media’s powerful and growing incorporation in clients’ daily lives [42, 45, 59, 70]. Clients are looking for social connections and social support, and want to learn more about treatment options and ways to achieve psychological wellbeing [36].

By applying the media content analysis method, methodologically the focus of analysis was the type of posted information without judging its richness or appropriateness. The interpretation of our results revealed the double-edged outcomes of clients’ search for such information and exchange in the domain of self-management when living in community settings. The bloggers’ political, educative and compassionate discourses were intertwined to create a mixed view of PC, especially with respect to interactions with physicians during and after treatment. These results are relevant to HCPs since they offer a better appreciation of the cultural meaning men attribute to PC and how men interpret a diagnosis of PC, as well as the emergence of experiential knowledge shared among PC survivors. For HCPs, the results suggest an area for future interventions to target deficits in men’s PC literacy so as to improve their understanding, self-care strategies, and potentially their PC trajectory.

This study’s findings, while based on data limited to mostly one-time blog entries, contribute to an understanding of men’s collective expression of lived experiences of PC, particularly their reactions, ways of coping, and thoughts on needed change. Beyond those who posted, an unknown number of men would have read the content and benefited (or not), without contributing themselves.

Regarding methodology, this study has three major strengths. First, the use of procedures of verification with HCPs and a French PC survivor in Paris to ensure that the analytical-interpretative framework suited the actual context of bloggers’ PC lived experiences. Second, the prolonged field engagement and concurrent other fieldwork-related research activities contributed to the study’s credibility and trustworthiness [66]. The results of a popular media analysis and a qualitative study based on conversations and interviews with PC hospitalized patients and survivors both contributed to an elaboration of the QOL framework we adopted. The combined fusion of all this data and findings reinforces this study’s credibility by virtue of providing referential adequacy material [66] and providing a holistic view of the cultural context of interest. Third, the participation of Canadian health experts (practitioner and researchers), acting as internal critics, confirmed the study’s empirical and theoretical soundness. The absence of any contact between researchers and the bloggers as informants can be interpreted as either a strength or a weakness; on the one hand, it implies a positive situational factor as a neutral form for data collection [82] that was uninfluenced by the researchers; on the other hand, it meant the bloggers could not be contacted to confirm meaning-in-context [83].

Regarding the study’s methodological weaknesses, the first is, again, the lack of contact with the bloggers to confirm our interpretation of their views and experiences of PC and their significance. The second weakness relates to the lack of verification and confirmation by informants, a critical concluding step in qualitative inquiry [66]. The third weakness is the absence of socio-demographic information on the bloggers, making it impossible to comment on potential group differences among the bloggers or between bloggers and non-bloggers. Altogether, these limitations compromise the findings’ transferability to another population.

Despite the potential bias and limitations of the study design, the results contribute to the public and professional debate about men’s capacity to provide emotional support and problem-solve through self-help collectives, information sharing, and advocacy via use of the Internet, an uncontrolled area for health professionals. Survivor groups are known to positively influence emotional management, information sharing, coping with decision-making process [33, 73], and PC literacy [71]. They also increase men’s and significant others’ engagement and, thereby, their ability to transcend limitations and improve QOL. Further studies should try to determine whether this sort of blog participation (and reading) helps men, and to what extent (for example, compared to a face-to-face/virtual group experience).

Studies should also explore if and how interaction between bloggers and HCPs who are responsible for answering questions and addressing issues could inform, influence, reinforce, reshape and/or change individual and collective views of PC. It would be interesting to explore how the relatives of PC bloggers view and experience PC, and contrast the two groups’ construction of PC. Furthermore, a future study that allows the researcher to interview PC bloggers about the benefits of blogging on their overall QOL would be fruitful. Finally, we suggest that future studies follow Koskan’s recommendation [45] to assess the clarity, readability, ease-of-usage, and credibility of blogs, and Hardiker’s [53] ideas for tailoring blogs to target population of users. The findings from such studies would help to establish standards of quality for blogs tied to the healthcare system.

## Abbreviations

HCPs: Health care professionals
PC: Prostate cancer
PSA: Prostate-specific antigen
QOL: Quality of life
